# Analyzing the Shortcomings in Smart Healthcare for Remote Home Care—A Case Study of the Taiwan Market

**DOI:** 10.3390/ijerph21070838

**Published:** 2024-06-27

**Authors:** Yunqi Yang, Grace T. R. Lin

**Affiliations:** Institute of Management of Technology, National Yang Ming Chiao Tuing University, Hsinchu 300, Taiwan; yangyunqi0077@gmail.com

**Keywords:** smart healthcare, telemedicine, home remote care, AHP (analytic hierarchy process), expert in-depth interviews

## Abstract

In Taiwan, remote healthcare initially focused on telemedicine, with systematic development starting in 2007 through the “Remote Care Pilot Program” and subsequent initiatives. Significant advancements came with the “Remote Health Care Services Development Plan” in 2010, which integratedArtificial intelligence and Information and communications technologies, enhancing smart healthcare in home care. This study investigated strategic gaps in smart healthcare applications for remote home care using a mixed-methods approach, particularly the Analytic Hierarchy Process (AHP). This study identified and ranked strategic gaps, including “legal regulations”, “economic factors”, “user behavior habits”, “policy and culture”, and “environment and technology”. Findings, based on in-depth interviews with 6 experts and 16 AHP questionnaire samples, highlight “legal regulations” and “user behavior habits” as critical areas needing attention. Addressing these gaps can improve user acceptance and the effectiveness of smart healthcare applications, providing valuable insights for future research and practice in making remote home care more comprehensive and efficient.

## 1. Introduction

### 1.1. Research Background and Goals

The year 2020 marked the beginning of commercial 5G deployment in Taiwan. Characterized by its high speed, high reliability, and low latency, 5G technology is regarded as a key driver for the integration of network platforms and the healthcare industry. As pointed out by Ge [1], these characteristics of 5G are instrumental in mitigating the deficiencies and imbalances in Taiwan’s healthcare resources development, especially considering the aging population issue that Taiwan is facing. The urgency for the application of smart healthcare becomes particularly evident. Furthermore, Sheng et al. [2] suggest that the advantages of 5G’s high speed, low latency, and high capacity should be fully utilized to facilitate the integration of 5G networks with smart mobile healthcare services, which will effectively address the core issues currently faced by the healthcare industry.

As the technology industry continues to progress and Taiwan’s population structure becomes increasingly aged, the traditional healthcare industry is no longer sufficient to meet the modern demands for medical services. As stated by Lv et al. [3], in response to this trend, traditional face-to-face medical services must shift towards telemedicine models. To address the long-term care needs of the elderly and the medical requirements of the patients with chronic diseases, the healthcare industry is widely adopting information and communication technologies to enhance the convenience of home-based remote care. This transformation indicates that Taiwan’s future healthcare service industry will broadly adopt new modes of service.

As the issue of aging intensifies, the increasing proportion of the elderly population makes elderly care a significant topic in contemporary society. Many geriatric diseases progress rapidly and require urgent treatment within a short window of time. Delays in detection and intervention can severely hinder timely treatment opportunities. Therefore, the early detection and prompt management of health issues are crucial for the well-being of the elderly. Against this backdrop, utilizing high-tech means such as 5G to achieve the real-time monitoring of the health status of the elderly and to provide timely feedback to medical institutions has become an effective strategy to safeguard the health of the elderly.

Additionally, the early detection of diseases and timely treatment not only effectively reduce medical costs but also help alleviate the pressure on government healthcare expenditures. Given the mobility challenges faced by the elderly, the application of smart healthcare technologies significantly lowers their difficulties in accessing medical resources. The low latency of 5G technology plays a crucial role in ensuring the timeliness of medical data transmission and the success rate of diagnosis and treatment. Moreover, by analyzing monitoring data and establishing health models to predict future health conditions, the mortality rate from age-related diseases can be effectively reduced, thereby extending the expected lifespan of society.

The home care model not only saves on manpower costs by enabling a few caregivers to efficiently manage many elderly individuals, but it also reduces the economic and time costs associated with travel. Additionally, this model helps alleviate the issue of the uneven distribution of medical resources across regions, particularly in sparsely populated or economically underdeveloped areas, thereby better addressing the care challenges faced by the elderly.

However, the application of smart healthcare technology also faces numerous challenges and issues. First are legal issues; remote healthcare cannot obtain comprehensive information as face-to-face consultations do, presenting potential risks that could lead to misdiagnosis or insufficient care. Therefore, laws need to further clarify the scope of practice and responsibility distribution in telemedicine. Secondly, economic factors must also be considered, such as defining whether the costs of care products are borne by individuals or paid by the government, and how to balance the costs of services. Lastly, the security and privacy of personal data are equally important, and there must be legal provisions to ensure the safety of these data. These issues are key to the research discussed herein, aiming to propose solutions to promote the development and application of smart healthcare technology.

### 1.2. Reasons for Selecting the Taiwan Market as a Case Study

The application of AI technology in the medical field is increasingly widespread, demonstrating significant advantages in diagnostic techniques, and has become a major trend in Taiwan’s healthcare market. Traditional Chinese medicine’s “observation, listening, questioning, and pulse-taking” methods now seem outdated compared to the precision and rigor of modern medical technology. AI has found an ideal application in the medical industry, with Taiwan currently utilizing various types of medical robots, including surgical robots, gastrointestinal inspection and diagnostic robots, rehabilitation robots, and other therapeutic robots. For instance, therapeutic robots have been instrumental in improving the quality of life for patients with Alzheimer’s.

In Taiwan, over 85% of the individuals aged 65 and above suffer from at least one chronic disease. Chronic diseases, defined as health conditions lasting more than three months, require continuous or long-term management. Remote care technology allows these patients to maintain optimal health, which for elderly patients means extended life expectancy and reduced time and effort spent on managing their conditions and frequent hospital visits. According to the American Association of Retired Persons, 80% of the seniors prefer to receive medical care at home [4].

With the widespread adoption of 5G networks and their integration with AI technology, along with the continuous advancement of smart healthcare technologies and the application of IoT physiological measurement devices, future medical services will achieve home-based long-term care through remote connections. This will establish a comprehensive care network that operates year-round, providing continuous and meticulous care for patients. This development trend indicates the vast potential of smart healthcare, significantly enhancing the efficiency and quality of medical services.

### 1.3. Research Question

With the rapid development of artificial intelligence (AI), its integration into everyday life and work has become increasingly widespread, garnering significant attention in both practical and academic circles. An increasing number of scholars are focusing their research on the development and applications of AI. The remarkable performance of AI in the medical field is widely recognized. 

Therefore, the research questions of this study are primarily as follows:

(a) In Taiwan, what are the key points to pay attention to in the application of smart healthcare in remote home care?
(b) How to improve these shortcomings?

### 1.4. Research Process

Section 1 analyzes and expounds on the research background and current situation of the topic; Section 2 focuses on the collection and review of the literature related to home-based remote care and smart healthcare, and defines them; Section 3 builds the research framework of the article based on the literature review, and proposes reasonable research methods; Section 4 conducts a survey on the current market of long-term remote care; Section 5 and Section 6 provide strategic suggestions based on the research results and analysis given by experts as the conclusion of this study, and also provide derivative suggestions for the future development of remote home care and smart healthcare.

## 2. Theoretical Reference

### 2.1. Definition and Trends of Smart Healthcare

Smart healthcare is defined by Li [5] as the trend of integrating advanced technologies such as the Internet of Things (IoT), cloud computing, and high-level analytics into existing medical processes. The application of these technologies in the field of smart healthcare is extensive, having propelled the market entry of remote healthcare, remote patient monitoring, integrated electronic medical record management systems, patient wearable devices, online medical consultations and appointments, and AI-assisted diagnostics. In this context, the prospects for the application of 5G in smart healthcare are shown to be exceptionally vast.

Global smart healthcare and artificial intelligence are continuously advancing, with both mutually supporting each other [6]. Due to the inherent uncertainties in medical behavior and processes, medical information technology and health information management can provide more precise and timely information to assist healthcare personnel in making clinical decisions [7]. Additionally, besides combining database sharing and integrated system matching to automatically provide decision-making information, it is also important to ensure that healthcare personnel from interdisciplinary teams can access correct and useful information on time [8].

In the future, the application of smart healthcare in home remote care will become more widespread, with a sharp increase in the demand for home IoT health terminals, and the interoperability of medical information will become more comprehensive. This indicates the potential of smart healthcare technologies and their pivotal role in the future medical field [9].

### 2.2. Application of Human Factors Engineering to Smart Medicine

Human factors engineering is the science of combining human factors and technological factors [10]. Its concept includes understanding and designing software from the perspectives of human factors (anatomy, physiology, psychology, etc.) and studying human–machine interactions in terms of hardware machines and environmental factors. It also considers issues such as personnel health, safety, and comfort, designing safe systems or tools to enhance work effectiveness and efficiency [11].

In the field of healthcare, medical errors or abnormal events are important issues in patient safety and medical quality management [12]. The integration of information technology tools with human factors engineering advantages helps reduce the likelihood of human errors and improves the efficiency and effectiveness of healthcare team members, thereby achieving the goal of patient safety [13].

In summary, human factors engineering can reduce the risk of human errors and improve system safety and effectiveness [14]. Therefore, to enhance medical quality and ensure the safety of healthcare personnel and patients, Taiwan is currently making great efforts to construct smart healthcare while designing, developing, or constructing various medical systems and devices. This involves referencing relevant human factors engineering standards and guidelines, consulting human factors engineering design experts, and designing medical equipment and systems that are safer, more efficient, and easier to use for elderly individuals.

### 2.3. Home Remote Care

Shi et al. [15] pointed out that home remote care is an essential component of the long-term care system, encompassing a variety of professional services, greatly facilitating the various issues faced by patients receiving care at home [16]. 

As Taiwan’s aging population continues to grow, the demand for long-term care is increasing [17]. Therefore, developing Tele-home Care (THC) has become a significant direction in smart healthcare. The primary advantage of THC is that it enhances the capabilities of family caregivers and improves the quality of life for the entire family [18]. For those care recipients who do not require daily medical assistance, THC facilitates convenient two-way interaction with professional medical personnel through wired or wireless transmission and equipped micro-physiological parameter sensors. This not only reduces the travel burden for both parties but also enhances the mobility and disease self-management of the care recipients [19].

This study focuses on the categories of home remote care, exploring the use of smart electronic medical devices and 5G communication technologies to enable elderly patients or their relatives to monitor health indicators such as blood pressure and blood sugar at home, and store data in real-time on device apps, further uploading it to the smart hospital center service platform. This process not only allows medical personnel to conduct timely remote disease detection and tracking but also greatly facilitates home patients, eliminating their need to visit hospitals or clinics.

### 2.4. The Shortcomings in Smart Healthcare for Remote Home Care

#### 2.4.1. Laws and Regulations

Taiwan’s regulations in the field of telemedicine were relatively strict until the outbreak of the COVID-19 pandemic, which saw them become more lenient. Similar to Japan’s approach of easing regulations through administrative interpretation, Taiwan also adjusted its “Regulations on Telemedicine,” but unlike Japan’s comprehensive opening strategy, Taiwan only expanded the scope of application, mainly targeting groups under home quarantine or isolation. Additionally, prior to 2018, Taiwan’s “Physician Act” stipulated that, except in remote areas, medical services in other areas required the physical presence of a physician. However, on 10 January 2018, the Ministry of Health and Welfare introduced the “Draft Regulations on Telemedicine,” further broadening the scope of telemedicine. This included “follow-up treatment within three months after discharge for acute inpatients” and “residents of residential long-term care facilities”, among other new applicable groups, significantly expanding the target audience for telemedicine services.

The rapid development of electronic technology and the rise of the ICT industry have facilitated the widespread use of technologies such as 5G, video transmission, Bluetooth, and wireless networks, providing technical support for real-time home remote care and treatment. However, according to the current “Physician Act,” telemedicine has not been fully opened to all the Taiwanese people and is still awaiting further policy adjustments.

Legal responsibilities associated with telemedicine are also complex. Remote physicians may only provide professional opinions without establishing a substantial physician–patient relationship, which is referred to as “remote consultation” rather than “telemedicine”. Once a physician–patient relationship is established, the lack of face-to-face examination, such as palpation or potential distortions in image transmission, introduces uncertainty into the determination of negligence liability. This uncertainty may become a psychological barrier for physicians resistant to opening telemedicine [20].

Moreover, when significantly opening telemedicine, other detailed issues need consideration, such as ensuring that medical actions are performed to apply for health insurance payments, which might require recording the entire process to prove informed consent. Additionally, if physicians prescribe medications, patients might still need to physically visit pharmacies to collect their prescriptions, which is not a complete solution for those with mobility issues. Thus, how to reasonably open online pharmacies and address the opposition from physical pharmacies is also a critical issue that needs to be resolved in telemedicine policies. The implementation of these measures will have a profound impact on the development direction and industry trends of telemedicine services in Taiwan.

#### 2.4.2. Economic Factors

Due to the intensifying trend of an aging population [21] and the chronic nature of diseases resulting from advances in medical technology, along with changes in family structures and a reduction in family caregiving functions, elderly individuals face significant difficulties accessing medical care due to reduced mobility or disability. This situation leads to a significant increase in medical costs for the elderly. At the same time, Cai [22] has noted that remote areas face severe issues with the uneven distribution of medical resources. In this context, the intervention of remote care becomes particularly necessary, but the high costs of remote care are not affordable for every family, especially as the level of service increases, which also raises the costs. Therefore, economic factors become a critical consideration for many families when choosing remote care services.

One of the primary issues facing remote care is the network connectivity of smart healthcare devices. Although 2020 marked the commercial debut year of 5G technology, there are still issues with unstable connections or interruptions under the 4G network environment. Additionally, each home smart healthcare device operates on its independent system, lacking necessary integration. While technological devices are advanced and visually appealing, their high costs and solutions that do not target core pain points limit their widespread adoption. Although 5G networks offer the possibility of real-time data transmission, their high communication costs also deter many families with limited financial resources.

If the remote care industry could nurture several teams with an ecosystem mindset to collaboratively create an interconnected working model, a new care paradigm could potentially emerge. This model would redefine the framework of remote care through technology and innovation, likely playing a significant role in enhancing care quality, reducing costs, and more broadly disseminating smart healthcare devices.

#### 2.4.3. Policy and Culture

In 2021, the head of Taiwan’s Central Health Insurance Administration under the Ministry of Health and Welfare announced that the year would be designated as the inaugural year for telemedicine health insurance payments, with an allocation of TWD 100 million to include telemedicine within the scope of health insurance coverage. Initially, this policy will cover ENT (Ear, Nose, and Throat), dermatology, emergency, and outpatient services in 50 townships and districts. In the emergency sector, the subsidy per patient varies from a minimum of TWD 507 to a maximum of TWD 2340 depending on the triage category; for outpatient teleconsultations, a uniform subsidy of TWD 500 is provided. Additionally, in 2021, the Health Insurance Administration evaluated the data flow and administrative processes of telemedicine and explored the possibility of integrating home services, long-term care, and telemedicine.

However, currently, only certain departments have opened up to the use of telemedicine, and not all types of diseases can receive medical services remotely. The future development of telemedicine within the health insurance system remains unclear, and whether it will be fully opened is still uncertain [23].

In the digital healthcare industry, the widespread adoption of mobile devices and IoT medical equipment has become an irreversible trend. However, these devices pose data security risks during the information collection and transmission processes. For example, data may be corrupted during transmission, tampered with by humans, or hacked via Bluetooth capabilities, all of which can pose serious threats to patients receiving remote care. Consequently, the U.S. Food and Drug Administration (FDA) has issued guidelines requiring IoT-enabled medical devices to have adequate data security protections. Despite this, the rapid development of mobile device technologies, primarily smartphone apps, and their short market lifecycle mean that manufacturers aiming to quickly bring products to market may not fully consider data security and privacy protection. Since 2015, Taiwan’s Ministry of Economic Affairs has implemented a voluntary security certification mechanism for apps, but further mandatory implementation could hinder this rapidly changing industry. Therefore, further development at the policy level is still needed.

From a cultural perspective, Taiwanese society typically discourages leaving the elderly or patients alone at home, as any malfunction in remote care systems could pose a direct threat to their safety. This cultural characteristic adds significant socio-psychological barriers that must be overcome for the implementation of remote medical services. Therefore, future policy-making and technological innovations must take these cultural factors into account to ensure the widespread acceptance and effective implementation of remote medical services.

#### 2.4.4. User Behavior Habit

Currently, user behavior habits have not fully transitioned from traditional face-to-face medical consultations to remote medical and care services. Although technological advancements allow for medical test results to be accessed via the internet or applications (apps), many patients still prefer to be notified personally by a physician and desire the opportunity to directly ask medical personnel related questions.

Additionally, users are highly concerned about the privacy protection of their personal data. While real-time access to medical health information facilitates medical decision making, it is also susceptible to hacker attacks. According to monitoring by the Ministry of Health and Welfare, Taiwan’s government medical information system can experience up to tens of thousands of cyberattacks each night. For example, Under Armour’s health application was hacked in February 2018, resulting in the leakage of approximately 150 million user records. In 2013, a hospital in Los Angeles was hacked and had to pay a significant amount of Bitcoin to ransom encrypted electronic medical records. In 2017, 47GB of medical data stored in an Amazon S3 Repository by a company was illegally accessed.

In the context of the digitalization of medical information, non-medical information and communication technology (ICT) companies may gain access to personal medical and health data. The HITECH Act, passed in the United States in 2009, established clear privacy and security regulations for collaborations between non-medical and medical institutions [24]. However, devices such as Apple Watch smartwatches and fitness trackers, as well as data transmission on cloud platforms, may collect substantial amounts of users’ physiological data without needing to cooperate with medical institutions, creating legal loopholes.

Medical devices are generally categorized into three or four risk levels for market review. Most health and medical apps are primarily used to record physiological information, but some apps claim to convert smartphones into medical devices such as stethoscopes or combine them with external devices for diagnosis or treatment. If the operation of these apps involves medical professionals, safety concerns are minimal; however, if they are entirely operated by users, errors in devices that periodically record physiological information could lead to diagnostic errors [25].

#### 2.4.5. Environment and Technology

The development of smart healthcare, while full of potential, also faces significant challenges. Firstly, it encounters issues related to standardization, accuracy, and the acquisition, storage, and processing of big data. Key questions include the following: Who has the right to benefit from this data? Which medical teams or top medical experts are needed to review this data? Moreover, the development of medical artificial intelligence relies on vast amounts of medical data, often facing high costs and poor data quality.

The development of smart healthcare information systems requires close collaboration between medical and technical professionals [26]. However, these two types of professionals differ in their characteristics: the former excels in patient communication, while the latter communicates with machines. In most cases, achieving deep communication between the two is challenging, yet their effective collaboration is crucial for the in-depth research and application of the technology.

Secondly, the high degree of specialization in healthcare and information technology, coupled with information asymmetry and the lack of a common language for deep communication, presents another challenge. While technology is crucial, it should not dominate the healthcare industry. Ignoring this point can lead to project failure.

The third challenge is discussing smart healthcare without the support of a reliable medical team. For example, there are over 500 “diabetes management platforms” available on the App Store, but very few are widely used by medical teams and patients. Even wearable devices, despite being purchased, have few continuous users. Without professional support and ongoing improvements from medical teams, these devices are easily abandoned by users.

Finally, the lack of a sustainable business model is a significant challenge. For instance, although telemedicine has played a positive role in improving care quality and preventing patient regret, the monthly fee for Changhua Christian Hospital’s telemedicine service is only TWD 500. This model is difficult to sustain. Patients often choose to stop paying for the service after experiencing noticeable short-term improvements, reflecting the issue that high-quality medical services in Taiwan’s healthcare system often do not receive corresponding economic returns. Ongoing operational challenges may lead to the discontinuation of many effective medical methods, ultimately harming patient interests.

## 3. Method

### 3.1. Research Framework

This study employed expert interviews and the Analytic Hierarchy Process (AHP) as the primary research tools. Firstly, the research theme was determined based on the research background and motivation. Subsequently, the relevant literature and information in this field were collected and reviewed. Through the in-depth analysis, synthesis, and integration of these materials, the theoretical framework and empirical foundation of the study were established.

On this basis, the first layer of the AHP method was used to extract key strategic sub-factors obtained from expert interviews. These sub-factors serve as the cornerstone for constructing the subsequent research model, providing a scientific basis for questionnaire design. Following this, experts in the field were invited to complete the questionnaires, and the proposed strategies were analyzed in depth to distill effective strategic recommendations.

### 3.2. First Layer of the AHP Structure

**Figure 1:**
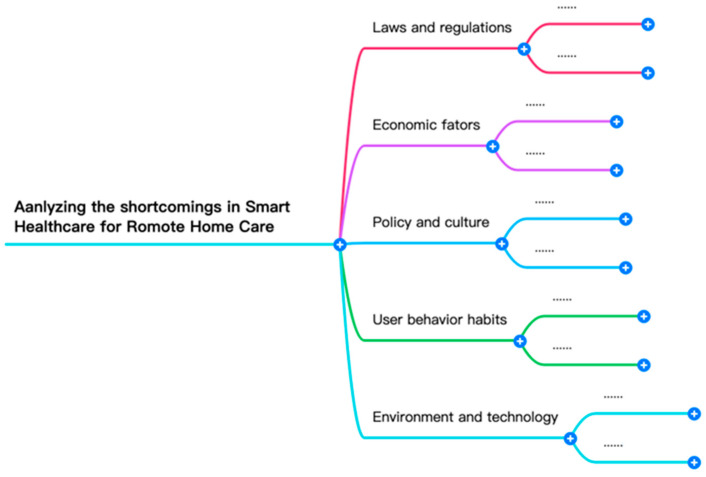
First layer of the AHP structure.

To facilitate the organization of this study, the AHP hierarchy structure includes the goal layer, criterion layer, and index layer, as shown in Figure 1. Additionally, this study further defines the five criterion layers as follows:

Laws and regulations (A);

Economic factors (B);

Policy and culture (C);

User behavior habits (D);

Environment and technology (E).

The corresponding third-level evaluation criteria are defined as A1, B1, C1, D1, E1, and so on.

### 3.3. Expert In-Depth Interviews

#### 3.3.1. Categories of Expert Interviews

Expert interviews can be categorized into three types based on their structure: structured interviews, semi-structured interviews, and unstructured interviews [27]. The definitions and characteristics of these three types of interviews are detailed below:

1. Structured Interviews: In this type of interview, the researcher designs a series of closed-ended questions in advance. During the interview, the researcher asks these predetermined questions one by one, and the interviewee responds based on pre-prepared answers. The main advantage of this method is the ease of recording and analyzing data. However, its limitation lies in the difficulty of obtaining in-depth and diverse insights, as the interviewee’s responses are often restricted and may not fully reflect their genuine thoughts.
2. Semi-structured Interviews: In this interview mode, the researcher designs a set of guidelines rather than specific closed-ended questions. Within this framework, the interviewee can freely express their views, and the researcher may ask new questions based on the interviewee’s responses for deeper exploration. This interview type maintains the focus of the discussion while offering sufficient flexibility, which helps improve the authenticity and validity of the data.
3. Unstructured Interviews: In unstructured interviews, the researcher does not set any closed-ended questions in advance, allowing the interviewee to freely express their true thoughts during the interview. The direction of the interview is guided by the interviewee’s immediate ideas and feelings. This interview format is highly beneficial for deeply exploring the interviewee’s personal experiences and genuine feelings, but it also requires more time and presents greater challenges in data organization and analysis.

#### 3.3.2. Reasons for Choosing Semi-Structured Interviews

Considering the three types of in-depth interviews, this study selected semi-structured interviews as the primary research method [28]. Following the literature review in Section 2, this study constructed the first layer of the AHP hierarchy to identify potential shortcomings or gaps in the application of smart healthcare in home remote care. Based on this framework, in-depth opinion interviews were conducted with scholars and experts in the relevant field.

The reasons for adopting semi-structured interviews are that this method maintains the focus of the research while providing sufficient flexibility, allowing interviewees to demonstrate the depth and breadth of their thinking in their responses. During the interviews, we posed the same questions to different experts, collecting both the differences and commonalities in their answers, and systematically categorizing and organizing the collected information. This approach not only enriches the content of the research but also provides solid data support for further in-depth studies.

#### 3.3.3. The Selection Process and Engagement of Experts

This study involved three rounds of expert interviews. The first and third rounds comprised the same group of six experts. Initially, from the first round of interviews, we derived the sub-criteria of the second tier of the AHP framework. Using these sub-criteria, we developed an AHP weight survey questionnaire, which was then administered to a second group of 16 experts. The results provided the weight and ranking of each factor. Subsequently, we conducted in-depth interviews with the same six experts from the first group in the third round to derive research strategies based on the study’s objectives. In total, 22 experts participated in this study. 

The six key experts who participated in the in-depth interviews are professors from the Institute of Biomedical Engineering at the National Yang Ming Chiao Tung University in Taiwan. Their specialized knowledge in smart healthcare and telemedicine significantly facilitated the smooth progression of the research. Their insights and expertise allowed for a comprehensive analysis of the shortcomings in smart healthcare for remote home care, leading to the formulation of strategic improvements. This multi-stage, multi-layered approach to expert engagement not only enhanced the reliability and validity of the research findings but also provided a novel methodology for future studies in the field of smart healthcare.

At the same time, all the interviewed experts have provided informed consent for participating in the research.

### 3.4. Analytic Hierarchy Process (AHP)

Based on the five application gaps identified from the literature review in Section 2, this study constructs the first layer of the Analytic Hierarchy Process (AHP) framework. To establish this framework and deepen the analysis, we first selected a group of technical experts, professors, and experienced practitioners with a strong background in smart healthcare and remote care for expert interviews. The purpose of these interviews was to gather their insights and evaluations regarding these gaps.

The expert interviews helped to identify the second-level indicators of the AHP framework, significantly enhancing the credibility of the study. Subsequently, using the Analytic Hierarchy Process (AHP), we designed an AHP questionnaire and distributed it to a second group of experts, different from those in the initial interviews. The questionnaire was scored based on specific AHP evaluation scales. Through the systematic analysis of the questionnaire data, we calculated the weights of each influencing factor in safety management, thereby objectively revealing the relative importance of each factor in risk management and compiling a comprehensive assessment report [29].

Finally, based on the questionnaire analysis results, we further consulted a third group of experts to discuss the management implications and gap strategy analysis reflected in the data. This process not only deepened the understanding of the application gaps in smart healthcare but also provided a scientific basis and strategic guidance for practical implementation.

The weight indicators of AHP are shown in Table 1.

### 3.5. AHP Execution Steps

**Figure 2:**
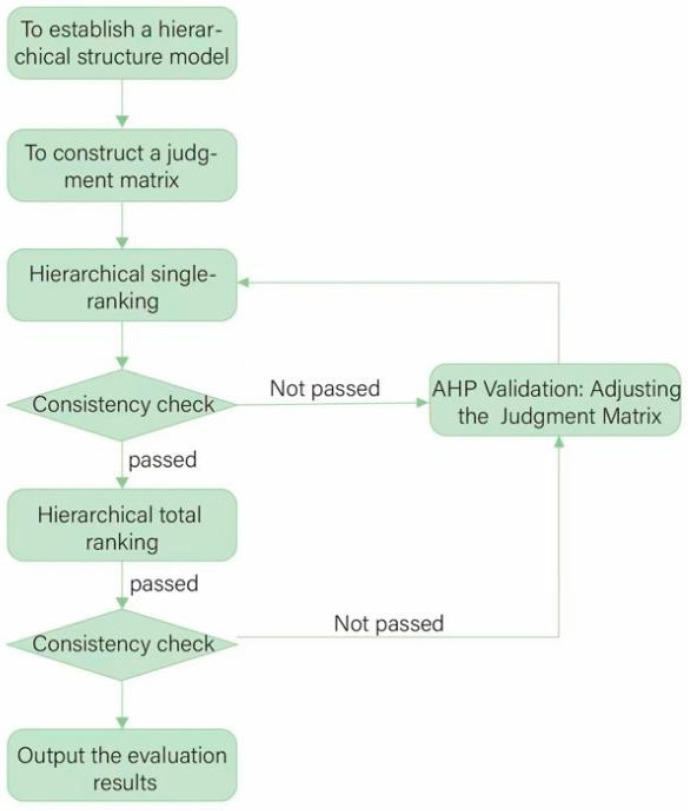
Execution steps of AHP.

Figure 2 shows the specific execution steps of AHP.

### 3.6. AHP Analytic Hierarchy Process Algorithm

After the establishment of the AHP matrix, it is necessary to calculate its vector value for weight. Saaty proposed the following four approximate methods to find the orientation value:

1. Line vector average standardization method

(1) $$W_{i}^{\prime }=\frac{1}{n}\sum_{j=1}^{n}\frac{a_{ij}}{\sum_{i=1}^{n}a_{ij}}$$

*i*, *j* = 1, 2, ……, *n*

2. The standardized method of column average

(2) $$W_{i}^{\prime }=\frac{\sum_{j=1}^{n}a_{ij}}{\sum_{i=1}^{n}\sum_{j=1}^{n}a_{ij}}$$

*i*, *j* = 1, 2, ……, *n*

3. The standardization method of row vector sum reciprocal

(3) $$W_{i}^{\prime }=\frac{\left(1/\sum_{i=1}^{n}a_{ij}\right)}{\sum_{j=1}^{n}\left(1/\sum_{i=1}^{n}a_{ij}\right)},$$

*i*, *j* = 1, 2, ……, *n*

4. Column vector geometric mean standardization method

(4) $$W_{i}^{\prime }=\frac{{\left(\prod_{j=1}^{n}a_{ij}\right)}^{\frac{1}{n}}}{\sum_{i=1}^{n}{\left(\prod_{j=1}^{n}a_{ij}\right)}^{\frac{1}{n}}},$$

*i*, *j* = 1, 2, ……, *n*

When calculating the vector value, the AHP method uses the first method to calculate the average value of row vectors. Because most of the matrices are inconsistent, the accuracy of this method is better. After calculating the vector, if you want to determine the consistency before and after, you need to calculate the *C.I.* value, and the formula is as follows:

(5) $$C.I.=\frac{\lambda -n}{n-1},$$

According to the formula, we need to find the *λ* value before calculating *C.I.*; therefore, using the weight w obtained above, we first calculate the consistency vector, which is represented by the *ν* symbol, so as to obtain the *λ* value. The formula is as follows:

(6) $$\nu_{i}=\left(\sum_{j=1}^{n}w_{j}a_{ij}\right)/w_{i},$$

*i*, *j* = 1, 2, ……, *n*

After the consistency vector is obtained, the *λ* value can be obtained by calculating the arithmetic average of its *ν* value, and the formula is as follows:

(7) $$\lambda =\frac{\sum_{i=1}^{n}\nu_{i}}{n},$$

*i* = 1, 2, ……, *n*

Finally, the *λ* value can be replaced by others to get the *C.I.* value; *C.I.* = 0 means that the judgment before and after is completely consistent, Saaty suggested.

When *R.I.* < 0.1, it can be regarded as having better consistency.

According to the research conducted by the Oak Ridge National Laboratory and Wharton School, the positive and negative matrices generated from evaluation scales 1–9 produce different *C.I.* values under different levels, which is called random index (random index; *R.I.*). The ratio of the *C.I.* value to the *R.I.* value is called consistency ratio (consistency ratio; *C.I.*) namely:

(8) $$C.R.=\frac{C.I.}{R.I.},$$

Therefore, Chen et al. [30] pointed out that the consistency of the matrix is very high when the value of *C.R.* is less than 0.1.

### 3.7. Related Work

The Analytic Hierarchy Process (AHP) is a widely utilized multi-level decision-making analysis method. Its primary advantage lies in its ability to decompose complex decision problems into several independent levels and sub-problems, thereby making the decision-making process more systematic and transparent. This method not only allows decision-makers to clearly understand and analyze issues at each level but also to integrate both subjective and objective factors, providing a more scientific and rational basis for decisions. 

Additionally, AHP is highly flexible, enabling decision-makers to easily simulate and analyze different decision-making scenarios, thereby facilitating more adaptive and effective decision making in the face of change and uncertainty. These strengths have led to the extensive application of AHP across various fields, particularly in complex decision-making scenarios that require the comprehensive consideration of multiple factors and the comparison of different alternatives.

## 4. Data Collection from Expert Interviews and Expert Questionnaires

### 4.1. AHP Second Index Layer

Based on the second-tier indicators of the five application gaps identified in the literature, the first batch of experts was interviewed. After the interviews, the unnecessary indicators were deleted, and the indicators deemed very important by the experts were added. The final indicators which were considered in the interview are shown in Table 2.

### 4.2. Data Compilation of Expert Questionnaires

Previous research indicated that when comparing two factors, the amount of content involved can influence the judgment of the respondents [31]. Generally, a range of 7 ± 2 is more suitable. We conducted a limitation at 9 and allowed expressing the difference between them on a scale of 1–9. When comparing, it is necessary to make *n*(*n* − 1)/2 pairwise judgments, providing more information and allowing for a more reasonable ranking through repeated comparisons across various aspects (Table 3). 

Table 3 shows the levels of importance and their respective numerical assignments used in the Analytic Hierarchy Process (AHP) to compare elements.

Random index (RI) values for the matrices of different orders, which are used in AHP to evaluate the consistency of pairwise comparison matrices. The RI values are determined in Table 4. Order 1 and order 2 showed RI = 0 due to the consistency of the positive reciprocal matrices.

### 4.3. Construction of Discriminant Matrix and Solution of Weight

Based on the index system and using the above-mentioned scaling method, a questionnaire survey was conducted using the expert consultation method. The second batch of 16 experts in this smart medical field were selected to independently score the importance of the index. The scoring results were discussed and summarized internally, resulting in the following pairwise discriminant matrix (Table 5).

The maximum characteristic root of the judgment matrix (*λ*_max_ = 5.0166) was calculated by the MATLAB software (Natick, Massachusetts, United States of America, 2024), and the average random consistency index (*RI* = 1.12) was determined by Table 4. In order to check the consistency of the judgment matrix, it is necessary to calculate the consistency index (*CI*) using the following formulation:

(9) $$CI=\frac{\lambda_{\max}-n}{n-1}=\frac{5.0166-5}{5-1}=0.0041,$$

The random consistency ratio (*CR*) was calculated by the following formulation:

(10) $$CR=\frac{CI}{RI}=\frac{0.0041}{1.12}=0.0037<0.10,$$

Therefore, it was concluded that the results of the AHP have satisfactory consistency, indicating that the distribution of weight coefficients was very reasonable. Moreover, the weight of the index was calculated by the MATLAB software, (Table 6).

For each index weight, we used the AHP to determine the index weight of each index layer. Construct judgment matrix was named as $S={\left(u_{ij}\right)}_{p\times p}$, and all the *CI* and *CR* were calculated by the formulations above.

Firstly, the *λ*_max_ of the indicators in laws and regulations was 3.0000, and the *CI* was 0 (Table 7). Based on *RI* = 0.58, *CR* = 0 was less than 0.1, it can be considered as a reasonable construction of the judgment matrix. The weight of each index layer was calculated and is shown in Table 8.

The *λ*_max_ of the indicators in user behavior habits was 3.0000, and the *CI* was 0 (Table 9). Based on *RI* = 0.58, *CR* = 0 was less than 0.1, it can be considered as a reasonable construction of the judgment matrix. The weight of each index layer was calculated and is shown in Table 10.

The λ_max_ of the indicators in user economic factors was 3.0092, and the *CI* was 0.0046 (Table 11). Based on *RI* = 0.58, CR was calculated as 0.0079, which was less than 0.1. Therefore, it was considered that the results of AHP have satisfactory consistency, and the distribution of weight coefficients was very reasonable. The weight of each index layer was calculated and shown in Table 12.

The *λ*_max_ of the indicators in policy and culture was 4.0211, and the CI was 0.0070 (Table 13). Based on *RI* = 0.9, CR was calculated as 0.0078, which was less than 0.1. Therefore, it was considered that the results of AHP have satisfactory consistency, and the distribution of weight coefficients was very reasonable. The weight of each index layer was calculated and shown in Table 14.

The *λ*_max_ of the indicators in policy and culture was 4.0042, and the *CI* was 0.00714 (Table 15). Based on *RI* = 0.9, CR was calculated as 0.0015, which was less than 0.1. Therefore, it was considered that the results of AHP have satisfactory consistency, and the distribution of weight coefficients was very reasonable. The weight of each index layer was calculated and shown in Table 16.

The integration of all the above contents, consistency ratios, and weights of the AHP criteria layer are presented in Table 17 and Table 18.

### 4.4. Reasons for Ranking Evaluation Criteria

In this study, the AHP questionnaire filled out by the second batch of experts was used to obtain the weight ranking of the application gaps of smart medical care for home remote care. Firstly, the study obtained preliminary results based on the literature survey. Then, the experts were asked why the current weight ranking was formed, which further established the depth and breadth of the study.

Through extensive literature investigation and analysis, this study identified five key application gaps and defined them as the five main criteria layers of AHP. After the first batch of experts reviewed and screened out some unnecessary evaluation indicators, each criterion layer included several evaluation indicators, totaling 17 indicators. Through quantitative analysis, the important application gaps were laws and regulations, user behavior habits, economic factors, policy and culture, and environment and technology.

Experts generally believe that laws and regulations are the most critical. At present, the Physician Law, Personal Information Protection Law, and the legal provisions on the quality and data responsibility of AI auxiliary equipment are not perfect. Legal norms are the basis for defining the legality of home remote care, so they are given priority.

Followed by user behavior habits, user acceptance directly affects the implementation of telecare and AI diagnosis and treatment. Especially for the elderly over 50 years old, they need to learn how to accurately upload their health data to the application so that doctors can diagnose and track them.

Economic factors ranked third. Although the government provides certain health insurance subsidies, especially for residents in remote areas, the residents in these areas may prefer community care to home remote care, because the elderly may feel lonely when they live alone, and they need interpersonal communication and social activities.

Policy and culture and environment and technology are relatively less important, mainly because these factors are greatly influenced by economic and environmental changes. Additionally, the government’s policy attitude and direction may change in the future.

## 5. Discussion 

### 5.1. Strategic Analysis of Laws and Regulations

In the post-pandemic era, experts generally believe that the “Physician Act” will gradually be fully opened, allowing physicians and patients to legally conduct remote diagnoses and treatments without the need for face-to-face interactions. This has become an inevitable development trend. Regarding how patients can obtain medications during home remote care, practices from abroad can be referenced: physicians prescribe medications for chronic disease patients through communication software, and patients then place orders on smart healthcare platforms, with the medications delivered directly to their doorstep. This method is both convenient and reduces unnecessary interpersonal contact.

During the telemedicine process, physicians can decide whether to use AI-assisted tools as needed. If any issues arise in home remote care, responsibility should be clearly defined: the decision-maker, usually the physician, should be accountable for the consequences of their decisions. However, experts also point out that when problems with AI-assisted tools themselves occur, the responsibility should fall on the device manufacturers, as this pertains to hardware issues; if errors in data lead to incorrect medical judgments, the data providers should be held accountable to address losses caused by software issues.

These discussions not only highlight the complexities faced by telemedicine in terms of legal, technical, and responsibility divisions but also emphasize the importance of future medical regulations, technical standards, and responsibility delineation. Ensuring the safety and effectiveness of home remote care requires systematic and detailed consideration of these critical factors.

### 5.2. Strategic Analysis of Economic Factors

Although the high cost of smart healthcare devices and 5G service fees cause many residents in remote areas to be hesitant about adopting home remote care, this care model can significantly reduce commuting and hospitalization costs. It is particularly beneficial for the continuous monitoring of patients’ health at home, thereby preventing higher treatment costs due to worsening conditions.

To address the economic gaps, this study proposes the following strategies:

1. Utilize information technology to establish an image exchange center to reduce unnecessary tests and examinations, thereby further lowering medical costs.
2. Combine big data analysis with targeted sampling reviews to curb redundant tests in the short term, including across different medical institutions.
3. Review the payment standards for tests and examinations item by item to prevent excessive fees.
4. Study the introduction of a partial co-payment mechanism for tests and examinations to alleviate the public’s financial burden.
5. To encourage large hospitals to transfer stable patients to other medical institutions, propose the following measures:
  - Compile rosters of medical institutions and departments in different regions of each county and city for reference by large hospitals when preparing patients for discharge.
  - Research incentive measures for large hospitals to refer patients.
6. Strive to maintain consistency in the competitive environment of regional hospitals.

Additionally, for remote areas, the government should collaborate with base station operators and medical institutions to provide greater subsidies, ensuring that the residents in these areas can benefit from home remote care. This policy should be distinct from those in metropolitan areas to ensure the fair distribution and effective use of resources. Such measures not only enhance the feasibility of home remote care but also improve the overall quality and efficiency of medical services in remote areas.

### 5.3. Strategic Analysis of Policy and Culture

Experts generally do not recommend fully integrating home remote care into health insurance coverage, primarily due to concerns that this may lead to service abuse, which could be detrimental to the healthy development of telemedicine services.

Culturally, it is advised to avoid leaving elderly or chronic disease patients living alone whenever possible. Additionally, it is essential to educate individuals under the age of 30 to enable them to guide the elderly in correctly using remote care applications (apps) and accurately uploading their health data. If an incorrect data upload leads to a misjudgment by the physician, the responsibility should be borne by the user. Such measures not only help improve the effectiveness of remote medical services but also enhance the users’ sense of responsibility towards the service.

The implementation of these strategies aims to enhance the quality and safety of remote care services while ensuring cultural adaptability and clear responsibility. This ensures that remote medical services improve convenience while maintaining their rigor and effectiveness.

### 5.4. Strategic Analysis of Users’ Behavior Habits

Firstly, strengthening information security is crucial. This includes implementing system firewalls, password protection, user authentication, and installing antivirus software and anti-hacker tools. Secondly, it is necessary to formulate or revise cybersecurity and medical service regulations related to telemedicine and provide the necessary education and training to users [32] to mitigate potential legal, ethical, and insurance issues.

Regarding public medical habits, the adoption of telemedicine technologies and solutions should be based on the suitability of the users and the mobility of the elderly. Young people with digital literacy should be encouraged to teach the elderly how to operate and learn skills such as online consultations. As this method involves establishing a new type of doctor–patient relationship, healthcare professionals will need to learn how to use and adapt to these new technologies to care for the elderly and chronic disease patients.

Moreover, addressing user concerns about personal information leakage can enhance trust through stronger user participation mechanisms. Additionally, users should be informed about the advantages of combining AI with home remote care. For example, smart healthcare systems can automate information management, ensuring that each patient receives appropriate attention and treatment, such as the regular monitoring and automatic adjustment of medication dosages. This not only improves healthcare quality and patient safety but also allows healthcare professionals to focus more on patient care rather than paperwork, reflecting the true value of smart healthcare.

The government has a responsibility to establish a secure review and monitoring system to protect the collective interests of the public and prevent the leakage of personal data. The continuity, comprehensiveness, interactivity, and timeliness of chronic disease remote care [32] make it an ideal choice, as chronic disease care requires continuous attention, and patients need to self-manage most of the time. Therefore, choosing home remote care not only meets patient needs but also effectively addresses the challenges of modern healthcare [33].

### 5.5. Strategic Analysis of Environment and Technology

This study identifies and analyzes the four major challenges facing smart healthcare and their corresponding solutions. Firstly, regarding the issue of “who should access health data and who needs to verify the data,” it is recommended that top medical teams and university medical faculty jointly take responsibility. Experts suggest lowering the cost of data acquisition and rigorously selecting high-quality data for research and patient treatment purposes.

The second challenge is the “high specialization in healthcare and information, leading to information asymmetry and a lack of a common language for deep communication”. To address this issue, it is essential to enhance communication between medical personnel and technical staff to eliminate information asymmetry. The government should implement subsidy measures to encourage collaboration between the industry, technical personnel, and doctors, thereby creating and meeting market demand and ensuring the quality and efficiency of medical services.

The third challenge is the “lack of reliable medical teams”. In Taiwan, this phenomenon is relatively rare, as the public can freely choose trusted medical teams, such as those from Veterans General Hospital or National Taiwan University Hospital. This choice should be elevated to a social communication level to enhance public trust in medical teams.

The final challenge is the “lack of a sustainable business model for smart healthcare”. Experts propose shifting from the traditional monthly fee model to a “smart hospital” service model, which continuously provides services to the public while achieving sustained profitability. This transition not only ensures the continuous provision of high-quality medical services but also guarantees the economic feasibility and long-term development of smart healthcare systems.

These strategies aim to address the main challenges in implementing smart healthcare, promoting its development towards greater efficiency and reliability through innovation and adjustment.

## 6. Conclusions

The results of this study provide valuable insights for the development of smart healthcare in Taiwan. With the increasing issue of an aging population, smart healthcare and remote medical care have become important approaches to alleviate pressure, enhance medical efficiency, and improve service quality. Taiwan, as a technologically advanced region, has a leading edge in the ICT industry and artificial intelligence technology, providing a solid foundation for the application of smart healthcare.

However, to realize the potential of smart healthcare, several challenges need to be addressed. Firstly, the incompleteness of regulations and policies may restrict the development and application of smart healthcare technology. Secondly, further research and guidance are needed on user acceptance and the usage habits of smart healthcare. Therefore, future research could focus on improving the regulatory and policy environment to promote the innovation and application of smart healthcare technology. Additionally, a deeper exploration of user needs and behaviors could provide smarter healthcare solutions that better meet practical requirements.

In addition to applications in smart healthcare, future research could also extend to the field of community care. With the intensifying aging population, community care has become an important supplementary approach. Therefore, researchers could explore how to utilize smart healthcare technology to enhance the quality and efficiency of community care services, better meeting the needs of the elderly.

Overall, smart healthcare and remote medical care have vast development prospects in Taiwan. However, realizing this potential requires joint efforts from academia, industry, and government to promote the innovation and application of smart healthcare technology, providing better guarantees for the health and happiness of Taiwan’s elderly population.

## Figures and Tables

**Table 1:** AHP evaluation scale.

Scale	Significance	Meaning
1	Equal importance	The two elements have the same importance.
3	Slightly important	One of the two elements is more important than the other.
5	Obviously important	One of the two elements is obviously more important than the other.
7	Strongly important	One of the two elements is much more important than the other.
9	Extremely important	One of the two elements is absolutely more important than the other.
2, 4, 6, 8	The compromise between the above two adjacent judgments.	A quantitative degree of compromise between the above two adjacent standards

**Table 2:** AHP hierarchical analysis method second-layer indicators.

Laws and regulations	Irrelevant telemedicine law guarantees the relationship between doctors and patients.
	Liability for medical negligence is not easy to divide.
	The standard for judging the behavior of health insurance payments is vague.
User behavior habits	Concerns about personal data disclosure.
	The information system APP fails to fully control and ensure information security.
	Inability to access timely medical quality.
Economic factors	Smart medical equipment has a high unit price.
	Requires a high income level.
	High network communication costs.
Policy and culture	Clinics are not fully integrated into medical insurance.
	Information security issues bear the risk of starvation.
	Conflicts with traditional ideas.
	The acceptance of smart medical applications among the elderly in biased areas is low.
Environment and technology	A sustainable business model is lacking.
	A reliable medical team is lacking.
	Cooperation between high-end information personnel and medical staff is needed.
	The acquisition, storage, and processing of big data.

**Table 3:** The 9 importance levels and their values.

Table Degree	Meaning
1	The Ci element and the Cj element have the same influence.
3	The Ci element has a slightly stronger influence than the Cj element.
5	The Ci element has a stronger influence than the Cj element.
07	The influence of the Ci element is obviously stronger than the Cj element.
9	The influence of the Ci element is absolutely stronger than the Cj element.
2 ,4, 6, 8	The ratio of the influence of the Ci element to the Cj element is between the above two adjacent levels.
1, 1/2, …, 1/9	The ratio of the influence of the Ci element to the Cj element is the reciprocal number above.

**Table 4:** RI values with matrix order 1–9.

Order	1 *	2 *	3	4	5	6	7	8	9
RI	0.00	0.00	0.58	0.90	1.12	1.24	1.32	1.41	1.45

*: *RI* < 0.05.

**Table 5:** Discriminant matrix of AHP index layer.

	Laws and Regulations	User Behavior Habits	Economic Factors	Policy and Culture	Environment and Technology
Laws and regulations	1	2	3	4	6
User behavior habits	1/2	1	2	2	3
Economic factors	1/3	1/2	1	1	2
Policy and culture	1/4	1/2	1	1	2
Environment and technology	1/6	1/3	1/2	1/2	1

**Table 6:** AHP index layer weight.

Index Layer	Weight
Laws and regulations	0.4416
User behavior habits	0.2339
Economic factors	0.1312
Policy and culture	0.1239
Environment and technology	0.0695

**Table 7:** Discriminant matrix of indicators of laws and regulations.

	Irrelevant Telemedicine Law Guarantees the Relationship between Doctors and Patients	Liability for Medical Negligence Is Not Easy to Divide	The Standard for Judging the Behavior of Health Insurance Payments Is Vague
Irrelevant telemedicine law guarantees the relationship between doctors and patients	1	3	3
Liability for medical negligence is not easy to divide	1/3	1	1
The standard for judging the behavior of health insurance payments is vague	1/3	1	1

**Table 8:** Weight of indicators of laws and regulations.

Index Layer	Weight
Irrelevant telemedicine law guarantees the relationship between doctors and patients	0.6000
Liability for medical negligence is not easy to divide.	0.2000
The standard for judging the behavior of health insurance payments is vague	0.2000

**Table 9:** Discriminant matrix of user behavior habits index layer.

	Concerns about Personal Data Disclosure	The Information System APP Fails to Fully Control and Ensure Information Security	Inability to Access Timely Medical Quality
Concerns about personal data disclosure	1	1	2
The information system APP fails to fully control and ensure information security	1	1	2
Inability to access timely medical quality	1/2	1/2	1

**Table 10:** Weight of user behavior habits indicator layer.

Index Layer	Weight
Concerns about personal data disclosure	0.4000
The information system APP fails to fully control and ensure information security	0.4000
Inability to access timely medical quality	0.2000

**Table 11:** Discrimination matrix of economic factors index layer.

	Smart Medical Equipment Has a High Unit Price	Requires a High Income Level	High Network Communication Costs
Smart medical equipment has a high unit price	1	2	3
Requires a high income level	1/2	1	2
High network communication costs	1/3	1/2	1

**Table 12:** Weight of economic factor indicators.

Index Layer	Weight
Smart medical equipment has a high unit price.	0.5396
Requires a high level of income.	0.2970
High network communication costs.	0.1634

**Table 13:** Discriminant matrix of policy and culture indicators.

	Clinics Are Not Fully Integrated into Medical Insurance	Risks of Capital Security Problems	Conflicts with Traditional Ideas	The Acceptance of Smart Medical Applications among the Elderly in Biased Areas Is Low
Clinics are not fully integrated into medical insurance	1	2	4	5
Risks of capital security problems	1/2	1	2	3
Conflicts with traditional ideas	1/4	1/2	1	2
The acceptance of smart medical applications among the elderly in biased areas is low	1/5	1/3	1/2	1

**Table 14:** Weight of policy and culture indicators.

Index Layer	Weight
Clinics are not fully integrated into medical insurance	0.5068
Risks of capital security problems	0.2641
Conflict with traditional ideas	0.1428
The acceptance of smart medical applications among the elderly in biased areas is low	0.0863

**Table 15:** Discrimination matrix of environmental and technical indicators.

	A Sustainable Business Model Is Lacking	A Reliable Medical Team Is Lacking	Cooperation between High-End Information Personnel and Medical Staff Is Needed	The Acquisition, Storage, and Processing of Big Data
A sustainable business model is lacking	1	2	2	5
A reliable medical team is lacking	1/2	1	1	3
Cooperation between high-end information personnel and medical staff is needed	1/2	1	1	3
Acquisition, storage, and processing of big data	1/5	1/3	1/3	1

**Table 16:** Weight of environmental and technical indicators.

Index Layer	Weight
A sustainable business model is lacking	0.4488
A reliable medical team is lacking	0.2346
Cooperation between high-end information personnel and medical staff is needed	0.2346
The acquisition, storage, and processing of big data	0.0819

**Table 17:** Consistency ratio and weight sorting of AHP criteria layer.

Criterion Layer	Evaluation Criterion	Consistency Ratio (CR)	Weight/Ranking
Laws and regulations (a)	A1–A3	0	0.4416/1
User behavior habits (d)	D1–D3	0	0.2339/2
Economic factors (b)	B1–B3	0.0079	0.1312/3
Policy and culture (c)	C1–C4	0.0078	0.1239/4
Environment and technology (e)	E1–E4	0.0015	0.0695/5

**Table 18:** AHP index data induction.

Primary Index	Weight	Secondary Index	Weight	Comprehensive Weight
laws and regulations	0.4416	Irrelevant telemedicine law guarantees the relationship between doctors and patients.	0.6	0.26496
		Liability for medical negligence is not easy to divide.	0.2	0.08832
		The standard for judging the behavior of health insurance payments is vague.	0.2	0.08832
User behavior habits	0.2339	Concerns about personal data disclosure	0.4	0.09356
		The information system APP fails to fully control and ensure information security.	0.4	0.09356
		Inability to access timely medical quality	0.2	0.04678
Economic factors	0.1312	Smart medical equipment has a high unit price.	0.5396	0.070796
		Requires a high level of income.	0.297	0.038966
		High network communication costs.	0.1634	0.021438
Policy and culture	0.1239	Clinics are not fully integrated into medical insurance.	0.5068	0.062793
		Risks of capital security problems.	0.2641	0.032722
		Conflicts with traditional ideas.	0.1428	0.017693
		The acceptance of smart medical applications among the elderly in biased areas is low.	0.0863	0.010693
Environment and technology	0.0695	A sustainable business model is lacking.	0.4488	0.031192
		A reliable medical team is lacking.	0.2346	0.016305
		Cooperation between high-end information personnel and medical staff is needed.	0.2346	0.016305
		The acquisition, storage, and processing of big data	0.0819	0.005692

## Data Availability

The author is willing to share all the data of the article publicly. If you need to obtain the data, please send an email to Y.Y. at yangyunqi0077@gmail.com.
